# A Multicenter Prospective Study on the Use of a Mandibular Advancement Device in the Treatment of Obstructive Sleep Apnea

**DOI:** 10.3390/dj11110247

**Published:** 2023-10-24

**Authors:** Eduardo Anitua, Pedro Mayoral, Gabriela Zamora Almeida, Joaquín Durán-Cantolla, Mohammad Hamdan Alkhraisat

**Affiliations:** 1 Sleep Unit, Clínica Eduardo Anitua, 01007 Vitoria, Spain; sleep@eduardoanitua.com (G.Z.A.); joaquin.durancantolla@gmail.com (J.D.-C.); 2 Regenerative Medicine Department, BTI Biotechnology Institute, 01007 Vitoria, Spain; mohammad.hamdan@bti-implant.es; 3 Sleep Unit, Clinica Bisheimer, 28006 Madrid, Spain; pedro.mayoral@pgoucam.com

**Keywords:** obstructive sleep apnea, mandibular advancement, mandibular advancement device, apnea–hypopnea index, respiratory polygraphy

## Abstract

The main objective of this prospective study was the evaluation of 1 mm step titration of mandible advancement in the success of treating obstructive sleep apnea (OSA). For that, a multicenter prospective study was designed to recruit patients with OSA who were eligible to receive a mandibular advancement device. Gradual titration of mandibular advancement (steps of 1 mm) from maximum intercuspidation was performed to determine the optimal mandibular advancement (highest reduction in the apnea-hypopnea index (AHI)). The principal variable was the percentage of patients where a reduction ≥50% of the AHI was achieved at the end of the titration phase. A total of 102 patients participated in this study. Fifty-six percent of the participants were males and 67% had a BMI ≥ 25 kg/m^2^. Most of the patients (79%) had an age ≥ 50 years and the majority (74%) were either non-smokers or ex-smokers. Excessive daytime sleepiness was reported by 40% of the patients. The mean AHI at baseline was 20.6 ± 12.7 events/h. The mean advancement of the mandible was 3.1 ± 1.6 mm. The device achieved a reduction in the AHI in 93% of the patients and success (≥50% reduction in the AHI) in 69% of the patients. Success was achieved in 50%, 81.6%, and 73.3% of the patients with mild, moderate, and severe OSA, respectively. Decreasing the magnitude of mandibular advancement could be possible by controlling the vertical mouth opening and step-by-step titration.

## 1. Introduction

The American Academy of Sleep Medicine (AASM) defines mandibular advancement splints as devices that are inserted into the mouth and modify the position of the mandible, tongue, and other upper airway support structures for the treatment of snoring and/or obstructive sleep apnea (OSA) [1]. In fact, in their recommendations, the American Academy of Sleep Medicine and the American Academy of Dental Sleep Medicine have established mandibular advancement device (MAD) as the treatment for snoring in adult patients who do not have obstructive sleep apnea (primary snoring) [2]. Mandibular advancement device is effective in reducing the apnea–hypopnea index (AHI) in adult patients with OSA and oxygen desaturation [2]. The mean reduction in the AHI is 13.60 events/h (95% confidence interval (95% CI): 15.57–12.20).

In dentistry, the prevalence of periodontitis in patients with OSA is four times higher than in the general population [3]. Patients with OSA have an increased clenching index and thus the likelihood of episodes of excessive occlusal loads [4]. Several studies have observed that signs of excessive loads like tooth wear, tooth fracture, and enamel cracks, have been associated with OSA [5,6]. The use of MAD acts as a splint with a protective effect against excessive loads that affect the integrity of tooth structure.

Mandibular advancement device has shown good long-term efficacy in the treatment of OSA [7]. Several studies have shown stability in AHI from 1 to 4 years in responding patients [8,9,10,11,12]. The improvement in the quality of life and the sleepiness symptoms has been also sustained over time [7,9,10]. Regarding blood pressure, a reduction in systolic and diastolic blood pressure measurements has been observed in one study after 2.5 to 4.5 years of starting treatment with MAD [10].

There are several factors that may influence efficacy, safety, and patient adherence to MAD [13,14,15]. The vertical mouth opening, the degree of mandibular advancement, and the stabilization of the position of the mandible during sleep are some of these factors. The first would increase the risk of backward falling of the mandible and thus stretching the upper airways. While the second may increase the likelihood of side effects (discomfort, alteration of occlusion, and adherence to treatment) [13,16,17]. And the third may increase the risk of losing, during sleep, the optimal position of the mandible.

In the treatment of sleep-breathing disorders with MAD, The Task Force of the AADSM (American Association of Dental Sleep Medicine) has termed the position of the mandible at which improvements in symptoms, signs, and objective indices are achieved as the appropriate therapeutic position [18]. During the titration of the mandibular advancement, the clinician searches for the position of the mandible at which the best subjective and/or objective results are achieved [17,18,19]. Furthermore, it should be the position that supports the highest probability of the patient’s adherence to the treatment and limits the secondary effects in the short- and long-term. Although several studies have shown that better improvement is observed at higher mandibular advancement [19,20,21], the individual analysis of the data indicated that some patients showed no improvement or even worsening of the AHI. Several studies have shown that no or small mandibular advancement has achieved an improvement in a group of patients [16,17,22,23]. For that, gradual titration of mandibular advancement is advisable and recommended to fine-tune the appropriate therapeutic position.

The higher the mandibular advancement, the lower the treatment adherence and the higher the risk of secondary effects [24,25]. Lee et al. in a finite element analysis study have shown that minimum and maximum restorative forces occurred at a mandibular advancement of 40% and 70% of maximum protrusion [26]. In another finite element analysis, Crivellin et al. have shown a positive relationship between mandibular advancement and the stress experimented on by the temporomandibular joint (TMJ) [27]. This observation has been also reported by Cohen-Levy et al. where almost a linear relationship existed between the mandibular advancement and the TMJ stress [28].

During the titration phase, the clinician needs to optimize the mandibular advancement and the vertical mouth opening to determine the most effective position in reducing the apnea–hypopnea index (AHI) and increasing the likelihood of adherence to treatment. In previous work, our research group suggested a gradual titration of mandibular advancement in 1 mm steps [17]. The titration has been performed considering the patient’s subjective symptoms and objective data of the apnea–hypopnea index. The study has been performed in 36 patients and has a retrospective design. Therefore, the main objective of this multicenter prospective study has been the assessment of MAD efficacy in treating OSA using the same 1 mm step titration protocol. The MAD has been digitally designed and digitally manufactured (CAD/CAM). The principal variable has been the percentage of patients who benefited from a reduction in the AHI by 50% or more at the end of the titration phase.

## 2. Materials and Methods

### 2.1. Study Design

This is a multicenter prospective study that was designed to assess the efficacy of the intraoral appliance APNIA^®^ (BTI Biotechnology Institute, Vitoria, Spain) in treating OSA, after being titrated gradually at steps of 1 mm of mandibular advancement. The patients were recruited between January 2014 and December 2020 in two private clinical centers in Spain (Eduardo Anitua Clinic and Clinica Bisheimer). All procedures involving human subjects were performed in accordance with the ethical standards of the institutional and/or national research committee and with the 1964 Helsinki Declaration and its later amendments or comparable ethical standards. The study was approved by the Ethical Committee of Investigation with Medicines of the Basque Country (FIBEA-03-EP/19/Eficacia DIA). All patients signed the informed consent.

The patients were included according to the following inclusion criteria:

- Adult patients (age ≥18 years).
- Patients were diagnosed with OSA.
- Patients accepted the treatment with the intraoral appliance APNIA^®^.
- Have complete records of the evolution of the AHI.
- Signed the informed consent.

The exclusion criteria were:

- Pregnancy
- Patients with TMJ disorders.
- Patients with active periodontal disease.
- Patients with less than 10 teeth per arch.

### 2.2. Study Procedures

An initial assessment and clinical history had been made of all patients. A series of intra- and extraoral photographs and radiographs were obtained. The aptitude of the patients to receive an intraoral appliance was assessed according to the criteria established in the clinical practice guide of the Spanish Sleep Society [29]. Patients were screened for having at least 10 teeth per dental arch, absence of teeth with infection, mobility or fracture, absence of prostheses with mobility or unstable elements, and absence of active periodontal disease.

Home respiratory polygraphy (BTI APNiA^®^, BTI Biotechnology Institute, Vitoria, Spain) was used for the diagnosis of obstructive sleep apnea and the titration of the mandibular advancement of the intraoral appliance.

The intraoral device was designed digitally using a three-dimensional model of the patient’s dental arch. The input data were acquired by intraoral scanning or extraoral scanning of study models. Using specific 3D modeling software, the intraoral appliance device (intraoral device APNiA^®^ (DIA), BTI Biotechnology Institute, Vitoria, Spain) was designed by adjusting and adapting the thickness of the splint according to tooth anatomy and biomechanical requirements. Vertical dimension was established in a digital articulator by increasing it to allow the insertion of the upper and lower splints (the minimal thickness required by the material is 0.5 mm for each splint) and considering the space between the most prominent molar cusp and the occlusal plane between both splints. For patients with deep bites, the increase in the vertical dimension was determined by adding the value of the overbite in millimeters to the 1 mm space for the upper and lower splints.

The devices were fabricated by additive manufacturing techniques (3D printing). For each splint, two metallic buttons (one on each side) were fixed on the lateral surface of the splint. The coupling mechanism allows lateral and protrusive movements, with a limited and controlled amount of mouth opening without retrusion of the mandible. During titration, 2 plastic retainers were connected to the ipsilateral buttons of the upper and lower splints. For all patients, the treatment was started at maximum intercuspidation with no advancement. After 2 weeks, the AHI was assessed with BTI-APNiA^®^. Then, the position of the mandible, when necessary, was brought forward in steps of 1 mm each. The appropriate therapeutic position was the position at which the maximum reduction in the AHI was achieved.

For the diagnosis and monitoring of obstructive sleep apnea, a sleep test was performed with a validated home respiratory polygraphy device (BTI APNiA^®^, BTI Biotechnology Institute, Vitoria, Spain). This device provided seven channels: respiratory flow, oxygen saturation, heart rate, body position, and snoring. It corresponds to classification III of the American Sleep Disorders Association (ASDA) and S3C4O1P2E4R2 of the SCOPER classification [30]. The sleep studies were analyzed automatically by BTI-APNiA^®^ software according to the criteria of the American Academy of Sleep Medicine [30,31]. The sleep analysis was monitored by a sleep technician and supervised by a sleep medicine specialist. To accept the sleep study, the minimum time of recording with good signals must be at least 240 min.

An apnea was defined as an event with a duration ≥10 s and a drop >90% (as compared to the baseline respiratory airflow) in the respiratory signal. Hypopnea was defined as an event when there was a decrease in the respiratory signal between 30% and 90% and a decrease in oxygen saturation ≥3%. The apnea–hypopnea index (AHI) was then calculated.

The value of the AHI was used to diagnose the presence and severity of the OSA. The patient had no obstructive sleep apnea if the AHI < 5 events/h. Mild OSA was diagnosed when the AHI was ≥5 events/h and less than 15 events/h. Moderate OSA was diagnosed when the AHI was ≥15 events/h and less than 30 events/h. Severe OSA was diagnosed when the AHI was ≥30 events/h.

### 2.3. Study Variables

The principal variable was the percentage of patients where a reduction ≥50% of the AHI was achieved at the end of the titration phase. The secondary outcomes were demographic and anthropometric data, social habits, Epworth sleepiness scale score, observed snoring, AHI, frequency of patients with AHI < 10 events/h, and mandibular advancement (mm).

### 2.4. Statistical Analysis

Mean and standard deviation were calculated for continuous variables. Frequency was calculated for qualitative variables. The normality of the distribution was assessed by the Shapiro-Wilk test for quantitative variables. Paired tests (Wilcoxon test or Kruskal–Wallis test) were used to compare the baseline and follow-up data. Categorical variables were compared by Chi-square test. Correlation analysis and Spearman correlation coefficient were calculated to identify factors that were correlated with the mandibular advancement on one side and with the MAD success on the other side. Statistical analysis was performed using SPSS 15.0 (SPSS Inc.; IBM; Chicago, IL, USA). Statistical significance was set at *p*-value < 0.05.

## 3. Results

One hundred and seventy-five patients were screened in the two participating centers. Seventy-three patients were excluded; 36 had not been diagnosed yet with obstructive sleep apnea, 30 had active periodontal disease, 7 had incomplete register of the AHI and 2 patients had TMJ disorders. Finally, 102 patients were enrolled in the study. The characteristics of the participants are described in Table 1. Fifty-five percent of the participants were males and 64% had a BMI ≥ 25 kg/m^2^. Most of the patients (79%) had an age ≥50 years and the majority (74%) were either non-smokers or ex-smokers.

The Epworth sleepiness score was less than 10 in 37% of the participants and excessive daytime sleepiness was reported by 40% of the patients. Moreover, snoring was reported by 69% of the patients. 

Table 2 shows the outcomes of the sleep study. The severity of the OSA varied between patients being mild in 38 (37.3%), moderate in 49 (48.0%), and severe in 15 (14.7%) patients. The mean AHI at baseline was 20.6 ± 12.7 events/h.

**Figure 1:**
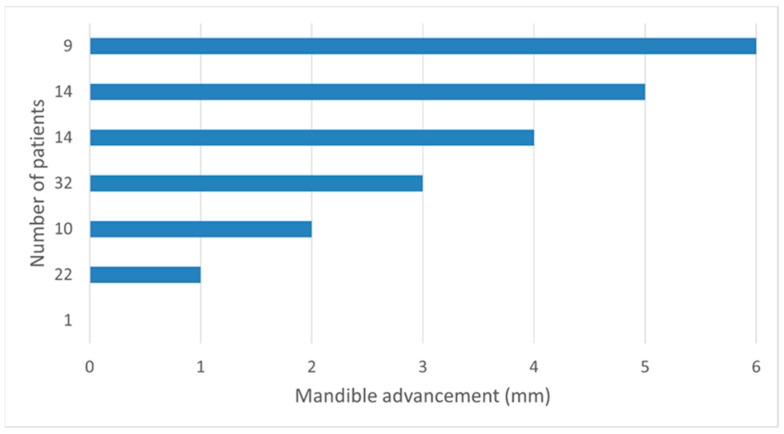
The degree of the mandible advancement at the end of the titration of the intraoral appliance.

The patients were treated by the intraoral appliance and the titration of the optimal degree of mandible advancement was performed in steps of 1 mm. Figure 1 presents a summary of the optimal mandible advancement. The mean advancement of the mandible was 3.1 ± 1.6 mm. Sixty-four percent of the patients required a mandible advancement ≤3 mm. Only 9% required an advancement of 6 mm. A correlation analysis was performed to identify factors correlated with the magnitude of mandibular advancement. A significant correlation was found between the mandibular advancement, the BMI, and the disease severity. However, the Spearman correlation coefficient was low for the correlation of mandibular advancement with BMI (0.294, *p*-value: 0.003) and disease severity (0.298; *p*-value: 0.002). It is worth mentioning that BMI and disease severity were also significantly correlated (Spearman correlation coefficient: 0.376; *p*-value: 0.000).

**Figure 2:**
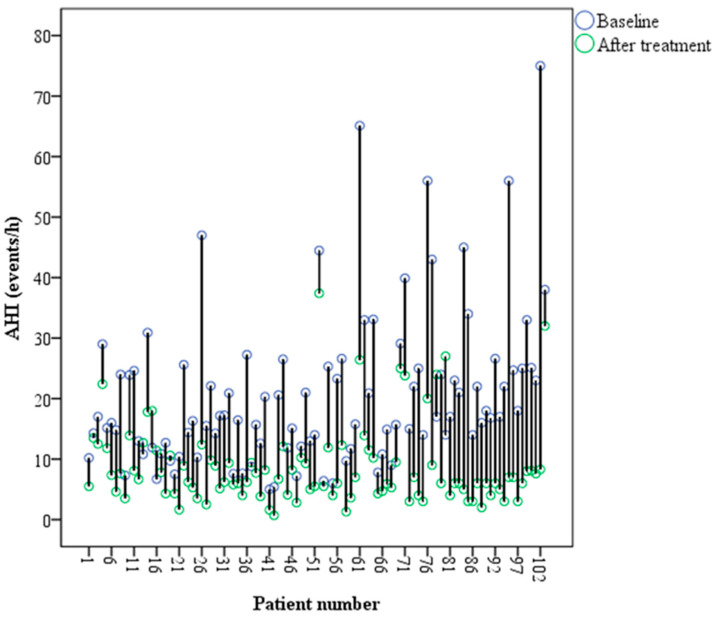
Apnea–hypopnea index at baseline and after treatment with the intraoral appliance.

The efficacy of the intraoral appliance in the treatment of obstructive sleep apnea is shown in Table 2 and Figure 2. The MAD had significantly improved the AHI, the supine AHI, and the non-supine AHI. A slight decrease (3.6%) in the sleep time in the supine position was observed.

Looking at the individual data of the AHI, 93% of the patients should an improvement in the AHI (Figure 2). The percentage of patients with AHI < 10 events/h was 15% before treatment and 75% after treatment. The mean unit reduction in the AHI was significantly affected by the severity of the OSA. Patients with more severe OSA achieved higher reductions in the AHI. Treatment success (reduction in the AHI ≥ 50%) was achieved in 69% of the patients. It was observed that the baseline AHI was statistically higher in the treatment success group (AHI of 22.5 ± 13.2 events/h vs. 16.4 ± 10.4 events/h, respectively). AHI worsening occurred in 6% of the patients. In those patients, the AHI was 11.3 ± 3.4 events/h at baseline and 16.2 ± 7.0 events/h after treatment. This increase in the AHI could be related in part to the night-to-night variability of the AHI.

Table 2 also shows that the intraoral appliance produced statistically significant changes in the severity of the OSA. The disappearance of OSA (AHI < 5 events/h) was achieved in 30.4% of the patients. According to the OSA severity, success was achieved in 50%, 81.6%, and 73.3% of the patients with mild, moderate, and severe OSA, respectively.

The characteristics of the patients were correlated to the success of the intraoral appliance. There was no significant association between appliance success and age or BMI.

## 4. Discussion

This study supports the use of mandibular advancement devices in the treatment of obstructive sleep apnea. The device has achieved a reduction in the AHI in 93% of the patients where 69% achieved a reduction ≥50%. This has been responsible for a statistically significant reduction in the severity of the OSA. More than 30% of the patients have been cured of OSA.

The titration protocols could be separated into two main groups where the outcomes should be verified by the physician [18]. In the first group, the titration is performed by only monitoring the improvement in the patient’s signs and symptoms. The second group combines the monitoring of the subjective measures with objective indices. Pulse oximetry, home-based respiratory polygraphy, or polysomnography have been included to support identifying the appropriate therapeutic position [18]. MAD titration in this study has been performed by monitoring the patient’s subjective feedback and respiratory polygraphy. The task force of the AADSM has indicated that the use of respiratory polygraphy needs education and to understand its contraindications (diagnosis or suspicion of insomnia, periodic limb disorder, central sleep apnea, narcolepsy) [18]. The titration protocol of this study has resulted in a mean mandibular advancement of 3.1 ± 1.6 mm. In more than 91% (93 patients) the mandibular advancement has been ≤5 mm and in 64% has been ≤3 mm.

The small mandibular advancement in this study could be related to the fact that the vertical mouth opening of the device has been controlled (limited to 2 mm). In order to avoid a decrease in the effective mandibular advancement. Other studies have reported higher mandibular advancement to achieve an effective reduction in the AHI [8,16,32,33,34,35]. One explanation could be the differences in the vertical mouth opening. Several studies have observed that increasing the vertical mouth opening may reduce the MAD efficacy [13,36,37]. The increase in the vertical mouth opening would induce a mandibular rotation in a posterior direction and would reduce the upper airway space. It has been estimated that for each 1 mm increase in the vertical mouth opening the MAD effectiveness in advancing the mandible is reduced by 0.3 mm [36].

The results of our study are in accordance with other studies supporting the efficacy of MAD in the treatment of OSA [38]. In a systematic review, an improvement in the AHI and patients’ symptoms has been observed in 92% of the patients treated with mandibular advancement devices [38]. In patients with AHI < 10 events/h or achieved AHI reduction ≥50%, the mean increase in the upper airway volume (with MAD in place) has been 1.95 cm^3^ (95% CI, 1.37–2.53; *p* < 0.001) [39]. The compartment with the higher increase has been the velopharynx. Moreover, the use of MAD has significantly improved the quality of life in patients with OSA [40]. Treatment with MAD may lead to a significant increase in the upper airway volume with a subsequent decrease in AHI. The velopharynx seems to be affected the most by OA therapy. Recent metanalysis of individual participant data has shown that mandibular advancement devices (titratable) were like CPAP in improving patients’ quality of life and sleepiness [41]. No differences have been observed between the two treatments in their effect on sleep structure. However, MAD has been superior to CPAP in preference and adherence to treatment [41]. The lower adherence to CPAP therapy may explain the absence of differences with MAD in relation to the quality of life, and functional and cognitive results [42]. CPAP has been associated with a higher decrease in the AHI and the oxygen desaturation index [41,43]. Additionally, there is evidence, although limited, that MAD may decrease the level of inflammatory cytokines (IL-1β, TNF-α, C-reactive protein) in blood [44]. MAD constitutes an alternative to CPAP in patients with mild and moderate OSA and patients with severe OSA but non-compliant with CPAP [42,45,46].

Moreover, custom appliances (like the one tested in this study) performed more favorably in the reduction in the AHI and have been used more hours per night compared to thermoplastic or readymade appliances [24,47,48]. The effectiveness of the MAD in treating OSA depends on patient adherence where comfort, side effects, and subjective enhancement of OSA interact [17]. All these findings emphasize the importance of the use of titratable MAD and refine the degree of mandibular advancement toward the least effective. The amount of mandibular advancement also affects the occurrence of MAD side effects [16].

In a systematic review, the MAD has resulted in dental and skeletal changes. The changes with statistical significance can be summarized in higher proclination of the lower incisors that resulted in a decrease in both the overbite and the overjet. A forward and downward rotation of the mandible and an increase in the angle between sella, nasion, and subspinale point A [25]. The changes in the occlusion have been also related to the time of MAD use and they have a progressive nature with the use time [49,50]. With a mean follow-up time of 11 years, dental changes have included mandibular crowding, decreased overbite, and overjet, increased intermolar and intercanine widths, posterior openbite, and anterior crossbite [50].

The limitations of this study included the absence of a control group and the short follow-up. Long-term follow-up is needed to assess device effectiveness, patient adherence, and the occurrence of skeletal/changes. The AHI has been measured by a validated respiratory polygraphy (type 3) which may provide uncertainty in the calculation of the AHI. The titration protocol may increase the need for more resources (clinical sessions, sleep studies) and time.

## 5. Conclusions

The use of mandibular advancement devices has been effective in the treatment of obstructive sleep apnea. Decreasing the magnitude of mandibular advancement could be possible by controlling the vertical mouth opening and step-by-step titration of it.

## Figures and Tables

**Table 1:** Patients’ characteristics.

Variable	Value
Age (years; mean ± SD ^1^)	58 ± 11
Sex (%)	46 females (45%); 56 males (55%)
BMI (kg/m^2^; mean ± SD)	26.7 ± 3.5
Smokers (number of patients; %)	12 (12%)
Epworth sleepiness score (mean ± SD)	7.6 ± 3.5
Snoring (number of patients; %)	70 (69%)
Excessive daytime sleepiness (number of patients; %)	41 (40%)

^1^ SD: Standard deviation.

**Table 2:** The efficacy of the intraoral appliance in the treatment of obstructive sleep apnea (OSA).

Variable			Value				*p*-Value	
AHI ^1^ (events/h) mean ± SD ^2^		Before treatment	20.6 ± 12.7				*p* = 0.000 ^3^	
		After treatment	8.6 ± 6.7					
Supine AHI (events/h) mean ± SD		Before treatment	34.8 ± 19.5				*p* = 0.000 ^3^	
		After treatment	13.8 ± 13.2					
Non-supine AHI (events/h) mean ± SD		Before treatment	11.7 ± 12.3				*p* = 0.000 ^3^	
		After treatment	5.7 ± 6.6					
Sleep time in supine position (%)		Before treatment	41.5 ± 23.8				*p* = 0.039 ^3^	
		After treatment	37.9 ± 23.6					
Reduction in the AHI (events/h) mean ± SD		Mild OSA ^2^	4.2 ± 5.1				*p* = 0.000 ^4^	
		Moderate OSA	12.7 ± 5.2					
		Severe OSA	29.3 ± 16.3					
Success (reduction in AHI ≥ 50%)			69%					
			**Severity OSA (after)**				**Total**	
			**No OSA**	**Mild**	**Moderate**	**Severe**		
Severity of OSA (before)	Mild	Number of patients	20	16	2	0	38	*p* = 0.000 ^5^
		Percentage	52.6	42.1	5.3	0.0	100	
	Moderate	Number of patients	9	37	3	0	49	
		Percentage	18.4	75.5	6.1	0.0	100	
	Severe	Number of patients	2	7	4	2	15	
		Percentage	13.3	46.7	26.7	13.3	100	
Total		Number of patients	31	60	9	2	102	
		Percentage	30.4	58.8	8.8	2.0	100	

^1^ AHI: apnea-hypopnea index. ^2^ SD: Standard deviation. ^3^ Wilcoxon test. ^4^ Kruskal–Wallis test. ^5^ Chi-square test.

## Data Availability

The datasets generated during and/or analyzed during the current study are available from the corresponding author upon reasonable request.
